# Medication and ECG Patterns That May Hinder SPECT Myocardial Perfusion Scans

**DOI:** 10.3390/ph16060854

**Published:** 2023-06-07

**Authors:** Marko Magdi Abdou Sidrak, Maria Silvia De Feo, Joana Gorica, Ferdinando Corica, Miriam Conte, Luca Filippi, Giuseppe De Vincentis, Viviana Frantellizzi

**Affiliations:** 1 Department of Radiological Sciences, Oncology and Anatomo-Pathology, Sapienza, University of Rome, 00161 Rome, Italy; 2 Department of Nuclear Medicine, Santa Maria Goretti Hospital, 04100 Latina, Italy

**Keywords:** ECG, myocardial perfusion scans, CAD, MPI

## Abstract

Coronary artery disease (CAD) is the leading cause of death followed by cancer, in men and women. With risk factors being endemic and the increasing costs of healthcare for management and treatment, myocardial perfusion imaging (MPI) finds a central role in risk stratification and prognosis for CAD patients, but it comes with its limitations in that the referring clinician and managing team must be aware of and use at their advantage. This narrative review examines the utility of myocardial perfusion scans in the diagnosis and management of patients with ECG alterations such as atrioventricular block (AVB), and medications including calcium channel blockers (CCB), beta blockers (BB), and nitroglycerin which may impact the interpretation of the exam. The review analyzes the current evidence and provides insights into the limitations, delving into the reasons behind some of the contraindications to MPI.

## 1. Introduction

Coronary artery disease (CAD) remains the leading cause of mortality in the United States (US), a trend that has persisted since 1990. CAD is responsible for a greater number of years of life lost due to premature mortality compared to the combined sum of lung, colon, breast, and prostate cancer. It is estimated that 10.9% of adults aged 45 or older and 17.0% of adults aged 65 or older in the US have CAD, respectively, with approximately 800,000 Americans experiencing a myocardial infarction (MI) each year. The economic burden of CAD is substantial, with estimated healthcare costs of $126.2 billion in 2010, which has been projected to increase to over $177 billion by 2040 [1,2,3]. Although there has been a decline in age-standardized mortality and years lived with disability (YLD) rates of CAD in the Middle East/North African region by 35% and 9%, respectively, over the past three decades, this region continues to bear a substantial burden of cardiovascular disease [4,5,6]. Age, uncontrolled blood pressure, high levels of cholesterol, diabetes, and tobacco use are all well-known risk factors for CAD. At present, hypertension is recognized as a significant, independent risk factor for coronary artery disease (CAD). Multiple studies have shown a consistent, progressive association between blood pressure and age-specific mortality from CAD, with higher blood pressure levels correlating with an increased risk of death from CAD, especially in individuals aged from 40 to 69 years old. In fact, a large meta-analysis found that a 20 mm Hg increase in systolic blood pressure, or a 10 mm Hg increase in diastolic blood pressure approximately doubles the risk of CAD-related mortality in this age group. Another study based on Framingham data revealed that blood pressure has the strongest association with cardiovascular disease (CVD) events in women and is the second most significant risk factor for men, surpassed only by age. Therefore, blood pressure is a crucial factor in the American College of Cardiology (ACC)/American Heart Association (AHA) Heart Risk Calculator, and blood pressure control is a fundamental component of the current AHA guidelines for the primary prevention of CVD. It is estimated that around 32.4% of US adults, equivalent to approximately 82 million individuals, have hypertension [7,8,9,10,11]. An economic analysis published by Bedetti et al. in 2008 on the costs of cardiovascular disease compared the relative cost-effectiveness of different cardiac imaging modalities. Stress echo imaging turned out to be 1.5 times more cost-effective than ex-ECG, while stress single-photon emission computed tomography (SPECT-Tc) was 3.1 times more costly, ex-ECG was 3.5 times more costly, cardiac troponin I (cTnI) was 3.8 times more costly, cardiac troponin T (cTnT) was 3.9 times more costly, and CA was found to be a staggering 56.3 times more expensive [12].

Myocardial perfusion scanning is crucial in both diagnosing and treating cardiac disease. These tests, which are non-invasive, help physicians to evaluate the blood flow to the heart muscle and obtain important information regarding the perfusion and metabolite uptake. This information is used to determine the most appropriate medical treatment or intervention to optimize cardiac health. Myocardial perfusion scans are valuable for diagnostic and prognostic purposes in various clinical settings, including evaluating angina symptoms, ruling out acute coronary syndrome as a cause of chest pain, assessing therapeutic outcomes after interventions, and determining viable or scarred myocardium [13]. Functional imaging with ^99m^Technetium-Sestamibi has been found to encompass a similar prognostic value compared to other methods. ^99m^Technetium-Sestamibi offers clear advantages over ^201^Thallium-SPECT, particularly when gated. Functional perfusion imaging has a clear advantage in predicting cardiac events [14]. In a study of 473 chest pain patients, those with normal scans had an extremely low annual mortality rate. Another review of 14 trials and over 12,000 patients with stable chest pain found that normal ^99m^Technetium-Sestamibi SPECT was associated with a hard cardiac event rate of 0.6% per year [15,16].

Among the used stressors, we commonly find adenosine and dobutamine with diagnostic and therapeutic applications in cardiac care. Adenosine is utilized in myocardial perfusion stress imaging studies due to its vasodilatory effects, while dobutamine, a sympathomimetic agent, affects the heart rate, blood pressure, and contractility. Adenosine’s pharmacological effects are mediated through purinergic adenosine receptors, while dobutamine selectively activates beta-1 receptors, enhancing contractility. Understanding the administration, effects, and indications of these agents is crucial for optimizing both cardiac assessment and treatment [17,18].

The purpose of this narrative review is to provide an opportunity to summarize the available evidence and provide a comprehensive overview of the current state of knowledge on the topic. By examining the utility of myocardial perfusion scans in relation to ECG alterations and medications, such as calcium channel blockers, beta-blockers, and nitroglycerin, as well as other conditions, this review can provide valuable insights into the clinical implications of these findings and the potential implications for patient care, while at the same time gaining insights on the procedural guidelines as for the reason of some of the contraindications [19].

## 2. ECG Alterations

### 2.1. Left Bundle Branch Block (LBBB)

LBBB, a frequent abnormality observed on electrocardiograms (ECGs), occurs when the normal conduction along the anterior and posterior left fascicles of the His–Purkinje system is disrupted. While LBBB is commonly linked to underlying heart conditions, and may arise from myocardial injury, strain, or hypertrophy, it can also manifest in patients presenting without specific clinical ailments [20]. LBBB is defined as a QRS duration equal to or greater than 0.12 s, and characterized by a broad-notched or slurred R wave in leads I, aVL, V5, and V6, an absence of Q waves in leads I, V5, and V6, and an R peak time exceeding 60 milliseconds in leads V5 and V6 but within the normal range in leads V1 to V3 [21].

**Figure 1:**
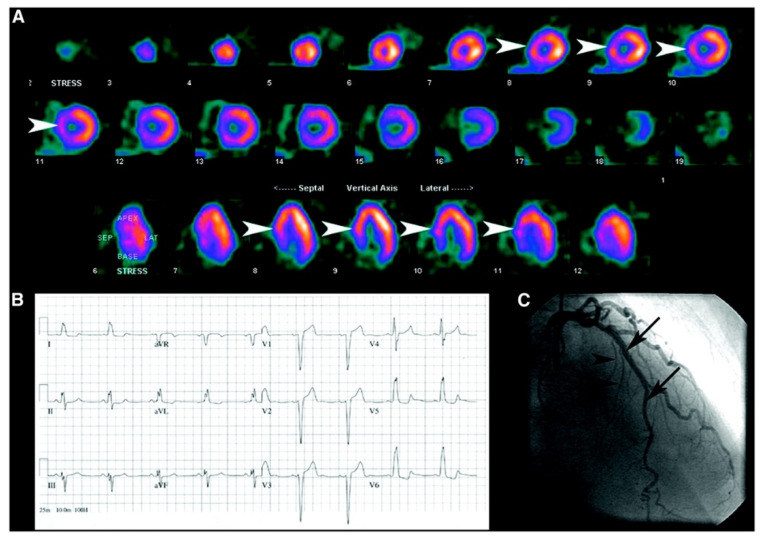
Patient with chest pain shows septal perfusion defect. (**A**) Myocardial perfusion scan obtained after injection of 99mTc-sestamibi during episode of pain shows septal defect (arrowheads). (**B**) Patient’s ECG reveals presence of LBBB. (**C**) Patient’s coronary angiogram reveals normal vessels supplying the septum, including left anterior descending artery (arrows) and septal perforators (arrowheads), indicating that septal defect was secondary to LBBB [26].

The ability of the noninvasive methods that are commonly employed, such as single-photon emission computed tomography (SPECT), to identify CAD in such patients is complicated by the diverse impacts of LBBB on the structure, function, and blood supply to the heart muscle. As a result, there is a considerable occurrence of anteroseptal and septal perfusion defects even in the absence of CAD (See Figure 1) [22,23]. A study by Larcos et al. involved 63 symptomatic patients with LBBB, 10 control subjects without LBBB and no CAD, and 10 normal control subjects. The participants underwent resting echocardiography and MCE (myocardial contrast echocardiography) and SPECT procedures. The study found that the septal wall/posterior wall (SW/PW) thickness and percentage thickening ratios were lower in the LBBB group compared to both control groups, but the resting SW/PW MBF (myocardial blood flow) and MBF reserve ratios were found to be similar across all three groups. MBF reserve was reduced in both the LBBB and control groups compared to the normal control group. SW thickness was a predictor of SPECT perfusion defects in LBBB patients without CAD. MCE was found to have a higher specificity for CAD detection compared to SPECT [24]. Another study was conducted to determine the diagnostic usefulness of myocardial perfusion imaging during exercise and pharmacological stress in patients with left bundle branch block. The study included 383 patients with left bundle branch block who underwent perfusion scintigraphy over five years. The study found that exercise, adenosine, and dobutamine tomography had a similar sensitivity and specificity for detecting >50% stenosis in the left circumflex and right coronary arteries. However, exercise tomography was found to have a higher false-positive rate for septal defects compared to the pharmacologic methods. The specificity and predictive value of a positive test response for left anterior descending coronary artery stenosis was higher for adenosine and dobutamine tomography compared to exercise tomography. Therefore, pharmacologic stress using adenosine or dobutamine tomography is more specific than exercise tomography in diagnosing left anterior descending coronary artery stenosis in patients with left bundle branch block. There was no significant difference observed between adenosine and dobutamine tomography in these patients [25] (see Table 1).

### 2.2. Atrioventricular Block (AVB)

The electrical impulse generated by the sinoatrial node must pass through the atria, the atrioventricular node, and the His–Purkinje system to reach the ventricles and trigger ventricular contraction, which is reflected as the PR interval and QRS complex on an ECG. If there is a delay in conduction within this system, this can result in an atrioventricular conduction block, leading to a prolonged PR interval on the ECG [27,28,29]. Atrioventricular (AV) conduction can be evaluated by assessing the relationship between the P waves and the QRS complexes. An AV block represents a delay or disturbance in the transmission of an impulse from the atria to the ventricles. This can be due to an anatomical or functional impairment in the heart’s conduction system. In general, there are three degrees of AV nodal blocks: first-degree, second-degree (Mobitz type 1 or 2), and third-degree [29,30,31]. Mobitz type I (Wenckebach) AV block is characterized by a progressive prolongation of the PR interval until an atrial impulse is completely blocked, resulting in a dropped beat on the ECG. Mobitz type II, on the other hand, has a constant PR interval for conducted beats, but also displays the occasional non-conduction of these atrial impulses without PR prolongation [32]. In third-degree, or complete, heart block, there is a complete absence of atrioventricular (AV) nodal conduction, resulting in no relationship between the P waves and the QRS complexes on the electrocardiogram (ECG). This means that the supraventricular impulses generated in the atria do not conduct to the ventricles [33]. A meta-analysis by Andrikopoulou et al. was the first to compare the rates of de novo atrioventricular block (AVB) and sinoatrial (SA) node dysfunction in patients undergoing pharmacologic single-photon emission computed tomography myocardial perfusion imaging (SPECT-MPI) with adenosine or regadenoson. The study found that the overall AVB incidence rate in patients using vasodilator stress SPECT-MPI was about 4%, and the high-grade AVB incidence rate was around 2%, respectively. Moreover, the incidence rates of overall and high-grade AVB were found to be significantly higher with adenosine than with regadenoson. None of the studies using regadenoson reported SA node dysfunction. Age and diabetes history were found to not be associated with the rates of de novo AVB [34]. Another paper evaluated the occurrence of AV block during adenosine stress testing in patients with first-degree AV block, with and without AV blocking medications. Of the 600 patients, 43 had a baseline PR interval >200 msec, and 557 had a baseline PR interval <200 msec. The study found that the frequency of second- and third-degree AV block during adenosine stress testing was significantly higher in patients with a baseline PR interval >200 msec than in patients with a normal baseline PR interval. However, the use of AV blocking medications did not increase the incidence of AV block during adenosine infusion. The study concluded that adenosine stress testing is safe for patients with baseline PR prolongation [35].

### 2.3. Balanced Ischemia

Balanced ischemia is a condition characterized by a relatively equal reduction in blood flow in all regions of the myocardium during stress. This can be due to various factors such as coronary artery disease, which may lead to the narrowing or blockage of the coronary arteries. Balanced ischemia is often identified by SPECT imaging as a uniform decrease in radiotracer uptake throughout the myocardium, indicating an impaired perfusion to the entire heart. As it is hard to tell apart from a normal heart scan, a positive stress ECG should alert the physician conducting the examination. Prompt recognition and management of balanced ischemia are crucial to prevent further complications and optimize cardiac function [36,37].

## 3. Medication

### 3.1. Calcium Channel Blockers (CCB)

Calcium, when it is in its ionized form (Ca2+), is widely recognized as a crucial second messenger in cells. The entry of Ca2+ into the cytoplasm is regulated by Ca channels, which allow Ca2+ to enter from external sources or be released from internal stores. Plasmalemmal ion channels, such as voltage-operated channels, receptor-operated channels, store-operated channels, and non-selective channels, are responsible for the entry of Ca2+ into the cell. Voltage-operated channels are further classified into three subtypes based on their conductance and voltage sensitivity, namely L (long-lasting), T (transient), and N (neuronal) subtypes. Calcium antagonists primarily block the entry of calcium into cells by targeting the L subtype of the voltage-operated channels, thereby altering their opening mode to favor the short-lived channels and shifting them to a long-lived closed state [38]. In clinical terms, calcium antagonists are categorized, based on their chemical composition, into three primary classes that are as follows: phenylalkylamines, which exerts a more pronounced influence on cardiac performance, resulting in negative chronotropic, dromotropic, and inotropic effects, but only modest vasodilation, dihydropyridines, which are potent vasodilators, and benzothiazepines, which fall in between in terms of cardiovascular impact [39,40]. Calcium-channel blockers are known to enhance myocardial perfusion by affecting myocardial microcirculation and metabolism. They have the potential to improve coronary flow in coronary artery disease (CAD) through selectively dilating larger arterioles. In addition to indirectly reducing myocardial oxygen demand through a decreased heart rate and contractility, calcium-channel blockers also directly decrease myocardial energy requirements. These drugs promote the utilization of free fatty acids in reversibly ischemic myocardium and mitigate myocardial stunning, regardless of hemodynamic changes [41,42]. Several studies have shown that the acute and chronic administrations of nifedipine and nicorandil improved perfusion and decreased ischemia in patients with CAD during exercise MPI. These CCBs were found to reduce the defect extent and severity, as well as the extent of ST-segment depression. Furthermore, previous research has also revealed that CCB in women presenting with chest pain and no family history of CAD could reverse a myocardial reversable perfusion defect in acetylcholine-positive angiograms [43,44]. Overall, the available data suggest that CCBs can improve myocardial perfusion during exercise in patients with CAD, although the effects on vasodilator MPI have not been studied. Similar conclusions can be drawn regarding the impact of CCBs on sensitivity of stress MPI as with BB [45,46,47].

### 3.2. Beta-Blockers (BB)

Beta-blockers are a class of drug that is used in order to reduce ischemic effects by lowering oxygen consumption during both rest and stress [48,49]. The catecholamines interact with the B1 receptors, leading to increased cardiac automaticity and conduction velocity, as well as renin release resulting in an elevated blood pressure. On the other hand, binding to B2 receptors induces smooth muscle relaxation and metabolic effects such as glycogenolysis. The effects of beta-blockers depend on their specificity towards different receptors and the organ system involved. Some beta-blockers may also bind to alpha receptors to varying degrees, resulting in different clinical outcomes under specific situations. Beta-blockers inhibit the effects of B1 and B2 receptors, leading to decreased heart rate and force of contraction. This in turn decreases blood pressure through reduced renin release and cardiac output. Beta-blockers also decrease oxygen demand by exerting negative effects on heart rate and contractility, thereby improving angina. Additionally, these medications prolong atrial refractory periods and exhibit antiarrhythmic effects as a result. Beta-blockers can be classified as non-selective or beta-1 selective. There are also beta-blocking drugs that selectively affect beta-2 and/or beta-3 receptors, but their clinical purposes are not yet known. Non-selective agents bind to both beta-1 and beta-2 receptors, resulting in antagonistic effects on both receptors. On the other hand, beta-1 receptor-selective blockers only bind to beta-1 receptors, making them cardio-selective [50,51,52]. Studies have shown a decrease in sensitivity in detecting coronary artery disease (CAD) in patients on BBs [53,54]. However, different doses, types, and modes of administration have been used to assess the effects of BBs on exercise or dobutamine MPI in the same patients while on or off BBs or in a cross-over or randomized, double-blind, placebo-controlled study design. Chronic administration of oral propranolol has been found to improve the tracer activity compared with the placebo or baseline studies without BBs, and intravenous propranolol administration to patients undergoing exercise MPI was also found to decrease the perfusion defect size compared with the placebo. Similar effects were observed in patients who underwent exercise or dobutamine single-photon emission computed tomography (SPECT) MPI. Acute propranolol administration before dobutamine MPI decreased the defect size and severity compared with the study without propranolol and normalized the scans in 23% of patients. The acute administration of metoprolol before dipyridamole MPI decreased the sensitivity of CAD detection and reduced the extent and severity of ischemia compared with the placebo. The effect of chronic atenolol use on dipyridamole SPECT MPI was assessed in a randomized, double-blind, cross-over study that revealed no significant differences in the perfusion defect size and severity between the placebo and atenolol for the group as a whole, although one-third of patients were found to display larger defects on atenolol than with the placebo [55,56]. BB, similar to CCB, have also found a role in positive acetylcholine angiograms in women presenting with chest pain [43].

### 3.3. Adenosine

As a therapeutic agent, adenosine is employed for its antiarrhythmic properties in supraventricular tachycardia (SVT), and it can also function as a diagnostic tool depending on the type of SVT. The pharmacological effects of adenosine are mediated through purinergic adenosine receptors, including the A1, A2a, A2B, and A3 receptors, which are distributed across various systems such as the immune, nervous, circulatory, respiratory, and urinary systems. Notably, the receptors located in the cardiac atrioventricular (AV) nodal tissue and peripheral vasculature exhibit clinical effects when adenosine is administered [57,58,59]. Adenosine causes vasodilation in the coronary system via A2a receptors, resulting in increased perfusion. However, this perfusion enhancement only occurs in the non-stenotic vessel segments. Post-stenotic vessel segments already exhibit the maximal dilation of resistance vessels through autoregulatory mechanisms to compensate for the pressure drop across the stenosis. As a result, vasodilators cannot further increase the perfusion in these areas. In myocardial SPECT, post-stenotic regions show lower uptakes of the radiopharmaceuticals compared to the normally perfused myocardial segments. Adenosine exerts additional effects through various adenosine receptors [60]. Adenosine induces a reflex increase in the heart rate by approximately 10% and a slight decrease in blood pressure due to vasodilation. Therefore, it does not impose a significant burden on the cardiovascular system, and myocardial oxygen consumption is only minimally increased. Unlike in exercise stress testing or dobutamine stress, adenosine rarely induces ischemia, except in cases of the coronary steal phenomenon. Adenosine has a short duration of action with a plasma half-life of <2 s, making it easily controllable. Symptoms and side effects of adenosine infusion are almost completely reversible within 1 to 2 min. Prolonged symptoms may indicate the possibility of vasospasm or acute coronary syndrome. Adenosine should be administered slowly to prevent a venous bolus, which may cause side effects such as a transient AV block and other bradycardias [61]. Adenosine is infused at a rate of 140 µg/kg/min over 6 min, and typically diluted in NaCl to a volume of 40 mL, resulting in an infusion rate of 400 mL/h. Shorter adenosine protocols with infusion times of 4 min, and radiopharmaceutical injection at 2 min are also possible [62]. Adenosine maximally increases myocardial perfusion to 3 to 4 mL/min/mg, which is higher than during exercise stress testing. However, SPECT radiopharmaceuticals show a plateau in uptake at perfusion values >2 to 2.5 mL/min/g. Therefore, despite the higher perfusion enhancements with adenosine, there are no fundamental differences in the uptake patterns and accuracy between exercise stress testing and pharmacological stress testing. PET perfusion radiopharmaceuticals have more favorable properties in this regard, allowing for absolute quantification and differentiation in high-flow situations, which is particularly relevant in the diagnosis of the early stages of coronary artery disease [63].

### 3.4. Dobutamine

Dobutamine, a sympathomimetic agent, increases heart rate, blood pressure, and contractility, leading to an increase in myocardial oxygen consumption and reflexive enhancement of perfusion, potentially provoking ischemia in the presence of coronary stenosis. The stress is administered in 3 min intervals, starting with an initial infusion rate of 5 µg/kg/min and gradually increasing to 10, 20, 30, and 40 µg/kg/min, respectively. The target heart rate to be achieved is (0.85 times (220-*age of the patient*)). If the target heart rate is not reached at the highest dose, atropine can be additionally administered (4 × 0.25 mg up to a maximum of 1 mg IV, with intervals of approximately 1 to 2 min). The target heart rate can be achieved in over 90% of patients. The effects of dobutamine, which has a plasma half-life of 120 s, can be countered with a beta-blocker. The criteria for terminating the test and contraindications align with those for exercise testing [18,64]. By selectively binding and activating beta-1 receptors in the myocardium, dobutamine increases contractility, thereby making it clinically indicated for decompensated congestive heart failure due to its sympathomimetic effects. The ionotropic effect of dobutamine enhances contractility, resulting in a decreased end-systolic volume and an increased stroke volume. This augmentation of cardiac output leads to an increase in the overall function of the heart [65,66,67].

### 3.5. Nitroglicerine

Nitroglycerin release nitric oxide, which leads to the production of cGMP which relaxes the smooth muscle cells, thereby preventing their contraction. This results in a marked relaxation of all components of the vascular system and a decrease in the pulmonary vascular pressure, intraventricular pressure, chamber size, and cardiac output [68]. Nitroglycerin also decreases coronary vascular resistance and increases the diameter of large conduit vessels, although the total coronary flow does not increase in the presence of obstructive CAD. Nitroglycerin selectively dilates microvessels distal to coronary stenosis, but myocardial perfusion remains constant. The anti-ischemic effects of nitroglycerin may also be due to the redistribution of the coronary flow from the normal to ischemic myocardium through the dilation of the collateral vessels [69,70,71]. Different doses and methods of administration of nitrates were used to evaluate their effects on myocardial perfusion imaging (MPI) using exercise SPECT [72,73]. The acute or chronic administration of nitrates have been found to decrease the severity or size of ischemic perfusion in the culprit zone compared to the placebo or baseline without nitrates. The improvement in perfusion was independent of changes in the heart rate or blood pressure and was mostly attributable to the enhancement of MBF. Treatment with nitroglycerin patches for four weeks was found to reduce the extent of ST-segment depression but did not influence perfusion defect severity [45,46,74,75].

## 4. Other SPECT Affecting Conditions

### 4.1. Hypertrophic Cardiomiopathy

Cardiomyopathies encompass a collection of diseases wherein the heart muscle itself is primarily affected. These conditions are categorized as ischemic or nonischemic, with nonischemic cardiomyopathies further classified as dilated, hypertrophic, or restrictive. Hypertrophic cardiomyopathy (HCM) is an inherited condition characterized by the presence of unexplained left ventricular hypertrophy, along with a non-dilated left ventricle and a preserved or an increased ejection fraction. Typically, the hypertrophy is uneven, with the basal interventricular septum showing the most pronounced thickening. Resting left ventricular outflow tract obstruction has been observed in approximately one-third of patients, while it was able to be induced in another one-third. In SEPCT imaging, it was found that it can disrupt the normal walls uptake, with the hottest pixels being in the septum, showing an apparent reduced perfusion in the rest of the left ventricle [26,76].

### 4.2. Dextrocardia

Dextrocardia is a rare cardiac congenital abnormality that presents itself with a right-sided heart mirroring normal anatomy [77]. Patients with dextrocardia exhibit distinct electrocardiogram (ECG) patterns. The standard 12-lead ECG in these patients reveal a significant right-axis deviation of the P wave and QRS complex. Lead I often shows a predominantly negative QRS complex and inverted P and T waves. Furthermore, the QRS complexes in leads aVR and aVL are reversed, resulting in a positive R wave in lead aVR. Additionally, there is a reversal of the usual QRS complex progression observed in the precordial leads, particularly in V4 to V6. By reorienting the precordial leads to a right-sided approach, specifically of leads V1 to V6, a characteristic pattern of septal depolarization and R-wave progression can be observed on the 12-lead ECG [78,79]. Various imaging modalities have been proposed for SPECT acquisition, including changing the arc of rotation and proning the position. The latter was deemed to potentially be the best suited option to keep the heart in the usual spot in the field of view, as well as reduce soft tissue attenuation [80,81].

### 4.3. Attenuation Correction

The presence of the pacemaker and LV leads in SPECT imaging may cause overestimations when applying CT attenuation correction (CTAC). However, a study found that the impact of these leads on SPECT findings was minimal. The overestimation was higher for ICD leads but remained at only 4%. The diameter of the leads and the composition of the materials used have been found to influence the levels of overestimation. Overall, CTAC was deemed useful despite the presence of these leads, as the leads did not significantly affect the SPECT results in both normal and high-resolution imaging. The study further clarified the limited impact of the leads on SPECT with CTAC [82].

## 5. Future Perspectives

**Figure 2:**
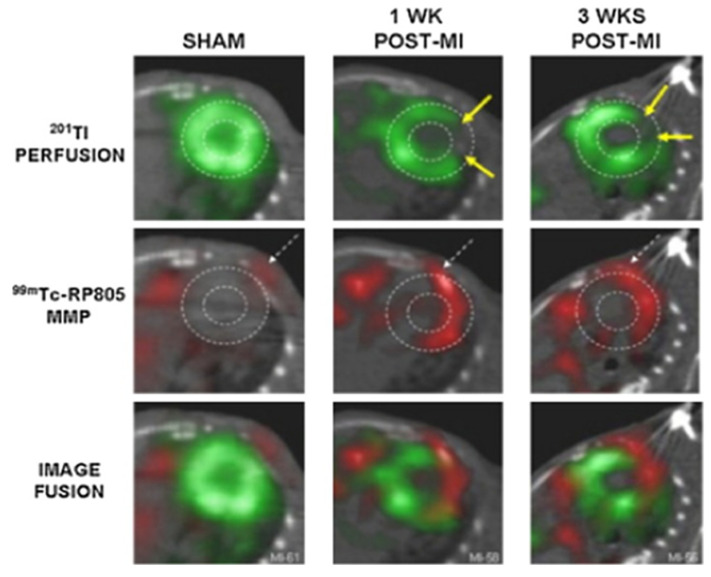
^201^Tl perfusion imaging, ^99m^Tc labelled MMP imaging, and fused images, 1 and 3 weeks after myocardial infarction using SPECT/CT [86].

Stress MPI has been conducted so far using perfusion tracers, such as MIBI, tetrofosmin, or ^201^Thallium. Functional abnormalities are yet to be studied with viability tracers such as ^18^F-FDG. The use of this tracer as a direct imaging agent for detecting exercise-induced myocardial ischemia shows great promise, as the differential glucose uptake between the normal and ischemic myocardium enables exercise 18 FDG to be used as a hot-spot imaging agent to detect this condition [83,84,85]. Other targets have also been proposed to evaluate post-infarcted myocardium, such as the extracellular matrix (ECM), particularly the metalloproteinases (MMP), which are involved in the degradation and remodeling of the heart. Angiogenesis has also been proposed as a target, with the development of tracer that binds α_v_β_3_, which is involved in endothelial cell survival and propagation and is present in response to angiogenic growth factors. Apoptosis has also been assessed using imaging, by labelling ^99m^Technetium to annexin V, a protein expressed on the cell surface of apoptotic cells (See Figure 2) [86].

ECG baseline abnormalities and ongoing medication will certainly play a role in imaging this tracer, and further studies will need to be conducted, as perfusion imaging will be challenged by ischemia imaging.

## 6. Conclusions

In conclusion, myocardial perfusion imaging is a useful diagnostic tool for patients with atrioventricular block (AV block) and left bundle branch block (LBBB) when pharmacologic stress is conducted, to evaluate the presence and extent of myocardial ischemia. It can also help clinicians in assessing the efficacy of medical therapies, such as calcium channel blockers, beta-blockers, and nitroglycerin, in reducing myocardial ischemia. While there is still ongoing research on the clinical significance of perfusion defects in these patient populations, the information obtained from myocardial perfusion scans can aid in guiding clinical decision-making and optimizing patient care, knowing when to evaluate a patient undergoing therapy, and when to stop therapy to assess their baseline heart condition. This procedure still presents a limitation put forward in a recent trial, where there was no indication that an initial invasive approach compared to a conservative one could lead to lower event rates or a lower overall mortality [87]. Therefore, it is important for clinicians to be familiar with the utility and limitations of myocardial perfusion imaging in the management of patients with these ECG alterations.

## Figures and Tables

**Table 1:** Summary table for the discussed ECG patterns, medications, and other condition that may affect SPECT imaging.

**ECG patterns**	**Left bundle branch block**	LBBB, a common abnormality on electrocardiograms (ECGs), can complicate the interpretation of SPECT images due to its diverse effects on the structure, function, and blood supply to the heart muscle. This can result in the presence of anteroseptal and septal perfusion defects even in the absence of coronary artery disease (CAD).
	**Atrio-ventricular block**	SPECT-MPI and AV block: A meta-analysis showed that the incidence of de novo AV block during pharmacologic stress single-photon emission computed tomography myocardial perfusion imaging (SPECT-MPI) with vasodilators including adenosine or regadenoson was around 4%, with a high-grade AV block incidence rate of approximately 2%. Adenosine was associated with a significantly higher incidence of AV block compared to regadenoson. Age and diabetes history were not associated with the rates of de novo AV block.
	**Balanced ischemia**	Balanced ischemia refers to a condition where there is an equal reduction in blood flow across all regions of the myocardium during stress. It is typically caused by factors including coronary artery disease, resulting in narrowed or blocked coronary arteries. SPECT imaging revealed a uniform decrease in radiotracer uptake throughout the myocardium, indicating impaired perfusion to the entire heart.
**Medication**	**Calcium channel blockers**	CCBs exhibit vasodilatory effects on larger arterioles, reducing myocardial oxygen demand indirectly through decreased heart rate and contractility. They also directly decrease myocardial energy requirements and promote the utilization of free fatty acids in the ischemic myocardium, resulting in a reduced defect extent, severity, and ST-segment depression.
	**Beta-blockers**	By exerting negative effects on heart rate, contractility, and renin release, beta-blockers improve angina symptoms and exhibit antiarrhythmic effects. Different types and doses of beta-blockers have been studied in exercise or dobutamine myocardial perfusion imaging (MPI), showing improvements in tracer activity and reductions in perfusion defect size and severity. Acute administration of beta-blockers before MPI has also been shown to decrease defect size and severity, while the chronic use of beta-blockers may not always result in significant differences in perfusion defect size and severity compared to the placebo.
	**Adenosine**	Adenosine causes vasodilation in the coronary system through A2a receptors, leading to increased perfusion. However, this effect is observed primarily in the non-stenotic vessel segments. Adenosine also induces a reflex increase in the heart rate and a slight decrease in the blood pressure. Importantly, adenosine stress testing rarely induces ischemia, except in cases of the coronary steal phenomenon. The duration of action of adenosine is short, and its side effects are reversible within minutes. Adenosine infusion should be administered slowly to prevent side effects such as transient AV block and bradycardias.
	**Dobutamine**	Dobutamine is a sympathomimetic agent that stimulates beta-1 adrenergic receptors, resulting in an increased heart rate, blood pressure, and contractility. It is commonly used in myocardial stress testing to evaluate myocardial viability and detect coronary artery disease (CAD). Dobutamine has a relatively short half-life, and its effects can be reversed with beta blockers if necessary. In addition to its diagnostic application, dobutamine is also used therapeutically in decompensated congestive heart failure to improve cardiac contractility and overall heart function
	**Nitroglycerin**	Nitroglycerin also reduces coronary vascular resistance and increases the diameter of large conduit vessels. The anti-ischemic effects of nitroglycerin may also be attributed to the dilation of the collateral vessels, redistributing the coronary flow from the normal to ischemic myocardium. Both the acute and chronic administration of nitrates have been shown to decrease the severity or size of ischemic perfusion in the culprit zone compared to the placebo or baseline without nitrates.
**Other conditions**	**Hypertrophic cardiomyopathy**	Hypertrophic cardiomyopathy is an inherited condition characterized by the unexplained thickening of the left ventricle, particularly in the basal interventricular septum. Around one-third of patients experience obstruction of the left ventricular outflow tract at rest or under induced conditions. In SEPCT imaging, HCM can disrupt normal wall uptake, resulting in reduced perfusion in most areas of the left ventricle except for the septum.
	**Dextrocardia**	Patients with dextrocardia show unique electrocardiogram (ECG) patterns, including right-axis deviation, inverted waves, and reversed QRS complexes. Reorienting precordial leads to a right-sided approach that allows for observing characteristic septal depolarization. Various imaging methods, such as changing rotation arc and prone positioning, have been suggested for SPECT acquisition to maintain heart visibility and reduce tissue attenuation.
	**Implantable devices**	The presence of pacemaker and LV leads in SPECT imaging may cause slight overestimation when using CT attenuation correction (CTAC). However, a study revealed minimal impact on SPECT findings, with only a 4% overestimation for the ICD leads. Lead diameter and material composition influence the level of overestimation. Overall, CTAC remained useful, and leads did not significantly affect the SPECT results, as clarified by the study.

## Data Availability

Not applicable.

## References

[1] Kochanek K.D., Xu J., Arias E. (2020). Mortality in the United States, 2019.

[2] Virani S.S., Alonso A., Aparicio H.J., Benjamin E.J., Bittencourt M.S., Callaway C.W., Carson A.P., Chamberlain A.M., Cheng S., Delling F.N. (2021). Heart disease and stroke statistics-2021 update: A report from the american heart association. Circulation.

[3] Odden M.C., Coxson P.G., Moran A., Lightwood J.M., Goldman L., Bibbins-Domingo K. (2011). The impact of the aging population on coronary heart disease in the United States. Am. J. Med..

[4] Dai H., Much A.A., Maor E., Asher E., Younis A., Xu Y., Lu Y., Liu X., Shu J., Bragazzi N.L. (2022). Global, regional, and national burden of ischemic heart disease and its attributable risk factors, 1990–2017: Results from the global Burden of Disease Study 2017. Eur. Heart J. Qual. Care Clin. Outcomes.

[5] Roth G.A., Mensah G.A., Johnson C.O., Addolorato G., Ammirati E., Baddour L.M., Barengo N.C., Beaton A.Z., Benjamin E.J., Benziger C.P. (2020). Global burden of cardiovascular diseases and risk factors, 1990–2019: Update from the GBD 2019 study. J. Am. Coll. Cardiol..

[6] Aminorroaya A., Yoosefi M., Rezaei N., Shabani M., Mohammadi E., Fattahi N., Azadnajafabad S., Nasserinejad M., Rezaei N., Naderimagham S. (2021). Global, regional, and national quality of care of ischaemic heart disease from 1990 to 2017: A systematic analysis for the global burden of disease study 2017. Eur. J. Prev. Cardiol..

[7] D’Agostino RBSr Vasan R.S., Pencina M.J., Wolf P.A., Cobain M., Massaro J.M., Kannel W.B. (2008). General cardiovascular risk profile for use in primary care: The Framingham Heart Study. Circulation.

[8] Goff D.C., Lloyd-Jones D.M., Bennett G., Coady S., D’Agostino R.B., Gibbons R., Greenland P., Lackland D.T., Levy D., O’Donnell C.J. (2014). 2013 ACC/AHA guideline on the assessment of cardiovascular risk: A report of the American college of cardiology/american heart association task force on practice guidelines. J. Am. Coll. Cardiol..

[9] Lewington S., Clarke R., Qizilbash N., Peto R., Collins R., Prospective Studies Collaboration (2002). Age-specific relevance of usual blood pressure to vascular mortality: A meta-analysis of individual data for one million adults in 61 prospective studies. Lancet.

[10] Rosendorff C., Black H.R., Cannon C.P., Gersh B.J., Gore J., Izzo J.L., Kaplan N.M., O’Connor C.M., O’Gara P.T., Oparil S. (2007). Treatment of hypertension in the prevention and management of ischemic heart disease: A scientific statement from the american heart association council for high blood pressure research and the councils on clinical cardiology and epidemiology and prevention. Circulation.

[11] Arnett D.K., Blumenthal R.S., Albert M.A., Buroker A.B., Goldberger Z.D., Hahn E.J., Himmelfarb C.D., Khera A., Lloyd-Jones D., McEvoy J.W. (2019). 2019 ACC/AHA guideline on the primary prevention of cardiovascular disease: A report of the american college of cardiology/american heart association task force on clinical practice guidelines. Circulation.

[12] Bedetti G., Pasanisi E.M., Pizzi C., Turchetti G., Loré C. (2008). Economic analysis including long-term risks and costs of alternative diagnostic strategies to evaluate patients with chest pain. Cardiovasc. Ultrasound.

[13] Patel J.J., Alzahrani T. (2023). Myocardial Perfusion Scan. [Updated 8 August 2022]. StatPearls [Internet].

[14] Gaudio C., Mirabelli F., Pelliccia F., Francone M., Tanzilli G., Di Michele S., Leonetti S., De Vincentis G., Carbone I., Mangieri E. (2009). Early detection of coronary artery disease by 64-slice multidetector computed tomography in asymptomatic hypertensive high-risk patients. Int. J. Cardiol..

[15] Soman P., Parsons A., Lahiri N., Lahiri A. (1999). The prognostic value of a normal Tc-99m sestamibi SPECT study in suspected coronary artery disease. J. Nucl. Cardiol..

[16] Iskander S., Iskandrian A.E. (1998). Risk assessment using single-photon emission computed tomographic technetium-99m sestamibi imaging. J. Am. Coll. Cardiol..

[17] Hallaj S., Mirza-Aghazadeh-Attari M., Arasteh A., Ghorbani A., Lee D., Jadidi-Niaragh F. (2021). Adenosine: The common target between cancer immunotherapy and glaucoma in the eye. Life Sci..

[18] Leppo J.A. (1996). Comparison of pharmacologic stress agents. J. Nucl. Cardiol..

[19] Verberne H.J., Acampa W., Anagnostopoulos C., Ballinger J., Bengel F., De Bondt P., Buechel R.R., Cuocolo A., van Eck-Smit B.L.F., Flotats A. (2015). EANM procedural guidelines for radionuclide myocardial perfusion imaging with SPECT and SPECT/CT: 2015 revision. Eur. J. Nucl. Med..

[20] Francia P., Balla C., Paneni F., Volpe M. (2007). Left bundle-branch block--pathophysiology, prognosis, and clinical management. Clin. Cardiol..

[21] Surawicz B., Childers R., Deal B.J., Gettes L.S., Bailey J.J., Gorgels A., Hancock E.W., Josephson M., Kligfield P., Kors J.A. (2009). AHA/ACCF/HRS recommendations for the standardization and interpretation of the electrocardiogram: Part III: Intraventricular conduction distur-bances: A scientific statement from the american heart association electrocardiography and arrhythmias committee, council on clinical cardiology; the american college of cardiology foundation; and the heart rhythm society. endorsed by the international society for computerized electrocardiology. J. Am. Coll. Cardiol..

[22] Tandogan I., Yetkin E., Ileri M., Ortapamuk H., Yanik A., Cehreli S., Duru E. (2001). Diagnosis of coronary artery disease with Tl-201 SPECT in patients with left bundle branch block: Importance of alternative interpretation approaches for left anterior descending coronary lesions. Angiology.

[23] Hirzel H.O., Senn M., Nuesch K., Buettner C., Pfeiffer A., Hess O.M., Krayenbuehl H.P. (1984). Thallium-201 scintigraphy in complete left bundle branch block. Am. J. Cardiol..

[24] Larcos G., Gibbons R.J., Brown M.L. (1991). Diagnostic accuracy of exercise thallium-201 single-photon emission computed tomography in patients with left bundle branch block. Am. J. Cardiol..

[25] Vaduganathan P., He Z.X., Raghavan C., Mahmarian J.J., Verani M.S. (1996). Detection of left anterior descending coronary artery stenosis in patients with left bundle branch block: Exercise, adenosine or dobutamine imaging?. J. Am. Coll. Cardiol..

[26] Burrell S., MacDonald A. (2006). Artifacts and Pitfalls in Myocardial Perfusion Imaging. J. Nucl. Med. Technol..

[27] Kashou A.H., Goyal A., Nguyen T., Chhabra L. (2022). Atrioventricular Block. StatPearls [Internet].

[28] Li X., Xue Y., Wu H. (2018). A Case of Atrioventricular Block Potentially Associated with Right Coronary Artery Lesion and Ticagrelor Therapy Mediated by the Increasing Adenosine Plasma Concentration. Case Rep. Vasc. Med..

[29] Batra A.S., Balaji S. (2019). Fetal arrhythmias: Diagnosis and management. Indian Pacing Electrophysiol. J..

[30] Saadi M., Tagliari A.P., Danzmann L.C., Bartholomay E., Kochi A.N., Saadi E.K. (2018). Update in Heart Rhythm Abnormalities and Indications for Pacemaker After Transcatheter Aortic Valve Implantation. Braz. J. Cardiovasc. Surg..

[31] Ali H., Furlanello F., Lupo P., Foresti S., De Ambroggi G., Epicoco G., Semprini L., Fundaliotis A., Cappato R. (2017). Clinical and electrocardiographic features of complete heart block after blunt cardiac injury: A systematic review of the literature. Heart Rhythm..

[32] Mangi M.A., Jones W.M., Mansour M.K., Napier L. (2023). Atrioventricular Block Second-Degree. [Updated 22 August 2022]. StatPearls [Internet].

[33] Kashou A.H., Goyal A., Nguyen T., Ahmed I., Chhabra L. (2023). Atrioventricular Block. 24 February 2023. StatPearls [Internet].

[34] Andrikopoulou E., Morgan C.J., Brice L., Bajaj N.S., Doppalapudi H., Iskandrian A.E., Hage F.G. (2019). Incidence of atrioventricular block with vasodilator stress SPECT: A meta-analysis. J. Nucl. Cardiol..

[35] Alkoutami G.S., Reeves W.C., Movahed A. (1999). The safety of adenosine pharmacologic stress testing in patients with first-degree atrioventricular block in the presence and absence of atrioventricular blocking medications. J. Nucl. Cardiol..

[36] Aziz E.F., Javed F., Alviar C.L., Herzog E. (2011). Triple vessel coronary artery disease presenting as a markedly positive stress electrocardiographic test and a negative SPECT-TL scintigram: A case of balanced ischemia. Heart Int..

[37] Lesser J.R., Bae R., Flygenring B., Sharkey S.S., Lindberg J., Schwartz R.S. (2005). Balanced myocardial ischaemia: A case of “normal” stress Tc99 sestamibi scan and diagnosis. Heart.

[38] Robertson R.M., Robertson D., Hardman J.G., Goodman Gilman A., Limbird L.E. (1996). Drugs used for the treatment of myocardial ischemia. Goodman and Gilman’s The Parmacological Basis of Therapeutics.

[39] Opie L.H. (1990). Clinical Use of Calcium Channel Antagonist Drugs.

[40] Grossman E., Messerli F.H. (2004). Calcium antagonists. Prog. Cardiovasc. Dis..

[41] Tillmanns H., Neumann F.J., Parekh N., Waas W., Möller P., Zimmermann R., Steinhausen M., Köbler W. (1991). Calcium antagonists and myocardial microperfusion. Drugs.

[42] Park S.W., Tang X.L., Qiu Y., Sun J.Z., Bolli R. (1996). Nisoldipine attenuates myocardial stunning induced by multiple coronary occlusions in conscious pigs and this effect is independent of changes in hemodynamics or coronary blood flow. J. Mol. Cell. Cardiol..

[43] Bugiardini R., Manfrini O., Pizzi C., Fontana F., Morgagni G. (2004). Endothelial Function Predicts Future Development of Coronary Artery Disease: A Study of Women with Chest Pain and Normal Coronary Angiograms. Circ. J. Am. Heart Assoc..

[44] Lanza G.A., Morrone D., Pizzi C., Tritto I., Bergamaschi L., De Vita A., Villano A., Crea F. (2021). Diagnostic approach for coronary microvascular dysfunction in patients with chest pain and no obstructive coronary artery disease. Trends Cardiovasc. Med..

[45] Stegaru B., Loose R., Keller H., Buss J., Wetzel E. (1988). Effects of long-term treatment with 120 mg of sustained-release isosorbide dinitrate and 60 mg of sustained-release nifedipine on myocardial perfusion. Am. J. Cardiol..

[46] Eldridge J.E., Burdick D.C., Jones R.H., Hossack K.F. (1987). Comparison of nitroglycerin patches and nifedipine. J. Cardiovasc. Pharmacol..

[47] Yamazaki J., Ohsawa H., Uchi T., Iida M., Nakano H., Hosoi H., Morishita T., Yabe Y., Koyama N., Komatsu H. (1993). Study of the efficacy of nicorandil in patients with ischaemic heart disease using ExerciseT1-201 myocardial tomography. Eur. J. Clin. Pharmacol..

[48] Guth B.D., Heusch G., Seitelberger R., Ross J. (1987). Mechanism of beneficial effect of beta-adrenergic blockade on exercise-induced myocardial ischemia in conscious dogs. Circ. Res..

[49] Koepfli P., Wyss C.A., Namdar M., Klainguti M., von Schulthess G.K., Lüscher T.F., Kaufmann P.A. (2004). Beta-adrenergic blockade and myocardial perfusion in coronary artery disease: Differential effects in stenotic versus remote myocardial segments. J. Nucl. Med..

[50] Gorre F., Vandekerckhove H. (2010). Beta-blockers: Focus on mechanism of action. Which beta-blocker, when and why?. Acta Cardiol..

[51] Rehsia N.S., Dhalla N.S. (2010). Mechanisms of the beneficial effects of beta-adrenoceptor antagonists in congestive heart failure. Exp. Clin. Cardiol..

[52] Machackova J., Sanganalmath S.K., Elimban V., Dhalla N.S. (2011). β-adrenergic blockade attenuates cardiac dysfunction and myofibrillar remodelling in congestive heart failure. J. Cell. Mol. Med..

[53] Hockings B., Saltissi S., Croft D.N., Webb-Peploe M.M. (1983). Effect of beta adrenergic blockade on thallium-201 myocardial perfusion imaging. Br. Heart J..

[54] Martin G.J., Henkin R.E., Scanlon P.J. (1987). Beta blockers and the sensitivity of the thallium treadmill test. Chest.

[55] Bridges A.B., Kennedy N., McNeill G.P., Cook B., Pringle T.H. (1992). The effect of atenolol on dipyridamole 201Tl myocardial perfusion tomography in patients with coronary artery disease. Nucl. Med. Commun..

[56] Taillefer R., Ahlberg A.W., Masood Y., White C., Lamargese I., Mather J.F., McGill C.C., Heller G.V. (2003). Acute beta-blockade reduces the extent and severity of myocardial perfusion defects with dipyridamole Tc-99m sestamibi SPECT imaging. J. Am. Coll. Cardiol..

[57] Samsel M., Dzierzbicka K., Trzonkowski P. (2013). Adenosine, its analogues and conjugates. Postepy Hig. Med. Dosw..

[58] Mosqueda-Garcia R. (1992). Adenosine as a therapeutic agent. Clin. Investig. Med..

[59] Rankin A.C., Brooks R., Ruskin J.N., McGovern B.A. (1992). Adenosine and the treatment of supraventricular tachycardia. Am. J. Med..

[60] Zoghbi G.J., Dorfman T.A., Iskandrian A.E. (2008). The effects of medications on myocardial perfusion. J. Am. Coll. Cardiol..

[61] Bokhari S., Ficaro E.P., McCallister B.D. (2007). Adenosine stress protocols for myocardial perfusion imaging. J. Nucl. Cardiol..

[62] Pennell D.J., Mavrogeni S.I., Forbat S.M., Karwatowski S.P., Underwood S.R. (1995). Adenosine combined with dynamic exercise for myocardial perfusion imaging. J. Am. Coll. Cardiol..

[63] Hashimoto A., Palmer E.L., Scott J.A., Abraham S.A., Fischman A.J., Force T.L., Newell J.B., Rabito C.A., Zervos G.D., Yasuda T. (1999). Complications of exercise and pharmacologic stress tests: Differences in younger and elderly patients. J. Nucl. Cardiol..

[64] McNeill A.J., Fioretti P.M., el-Said S.M., Salustri A., Forster T., Roelandt J.R. (1992). Enhanced sensitivity for detection of coronary artery disease by addition of atropine to dobutamine stress echocardiography. Am. J. Cardiol..

[65] Elhendy A., Valkema R., Van Domburg R.T., Bax J.J., Nierop P.R., Cornel J., Geleijnse M.L., Reijs A.E., Krenning E.P., Roelandt J.R. (1998). Safety of dobutamine-atropine stress myocardial perfusion scintigraphy. J. Nucl. Med..

[66] Alhayek S., Preuss C.V. (2023). Beta 1 Receptors. 8 August 2022. StatPearls [Internet].

[67] Kislitsina O.N., Rich J.D., Wilcox J.E., Pham D.T., Churyla A., Vorovich E.B., Ghafourian K., Yancy C.W. (2019). Shock—Classification and Pathophysiological Principles of Therapeutics. Curr. Cardiol. Rev..

[68] Katzung B., Chatterjee K. (1998). Basic and Clinical Pharmacology.

[69] Habazettl H., Vollmar B., Christ M., Baier H., Conzen P.F., Peter K. (1994). Heterogeneous microvascular coronary vasodilation by adenosine and nitroglycerin in dogs. J. Appl. Physiol..

[70] Feldman R.L., Marx J.D., Pepine C.J., Conti C.R. (1982). Analysis of coronary responses to various doses of intracoronary nitroglycerin. Circulation.

[71] Kanatsuka H., Eastham C.L., Marcus M.L., Lamping K.G. (1992). Effects of nitroglycerin on the coronary microcirculation in normal and ischemic myocardium. J. Cardiovasc. Pharmacol..

[72] Aoki M., Sakai K., Koyanagi S., Takeshita A., Nakamura M. (1991). Effect of nitroglycerin on coronary collateral function during exercise evaluated by quantitative analysis of thallium-201 single photon emission computed tomography. Am. Heart J..

[73] Göller V., Clausen M., Henze E., Giesler M., Schmidt A., Kochs M., Hombach V. (1995). Reduction of exercise-induced myocardial perfusion defects by isosorbide-5-nitrate: Assessment using quantitative Tc-99m-MIBI-SPECT. Coron. Artery Dis..

[74] Lewin H.C., Hachamovitch R., Harris A.G., Williams C., Schmidt J., Harris M., Van Train K., Siligan G., Berman D.S. (2000). Sustained reduction of exercise perfusion defect extent and severity with isosorbide mononitrate (Imdur) as demonstrated by means of technetium 99m sestamibi. J. Nucl. Cardiol..

[75] Mahmarian J.J., Fenimore N.L., Marks G.F., Francis M.J., Morales-Ballejo H., Verani M.S., Pratt C.M. (1994). Transdermal nitroglycerin patch therapy reduces the extent of exercise-induced myocardial ischemia: Results of a double-blind, placebo-controlled trial using quantitative thallium-201 tomography. J. Am. Coll. Cardiol..

[76] Marian A.J., Braunwald E. (2017). Hypertrophic Cardiomyopathy: Genetics, Pathogenesis, Clinical Manifestations, Diagnosis, and Therapy. Circ Res..

[77] Evans W.N., Acherman R.J., Collazos J.C., Castillo W.J., Rollins R.C., Kip K.T., Restrepo H. (2010). Dextrocardia: Practical clinical points and comments on terminology. Pediatr. Cardiol..

[78] Tanawuttiwat T., Vasaiwala S., Dia M. (2010). Mirror mirror (ECG image of the month). Am. J. Med..

[79] Mozayan C., Levis J.T. (2019). ECG Diagnosis: Dextrocardia. Perm J..

[80] Pawar S.U., Shetye S.S., Ghorpade M.K., Azeez Seena R. (2020). Assessment of Myocardial Viability Using Nuclear Medicine Imaging in Dextrocardia. J. Nucl. Med. Technol..

[81] Stathaki M., Koukouraki S., Papadaki E., Tsaroucha A., Karkavitsas N. (2015). The Benefits of Prone SPECT Myocardial Perfusion Imaging in Reducing Both Artifact Defects and Patient Radiation Exposure. Arq. Bras. Cardiol..

[82] Suzuki A., Koshida K., Matsubara K. (2014). Effects of pacemaker, implantable cardioverter-defibrillator, and left ventricular leads on CT-based attenuation correction. J. Nucl. Med. Technol..

[83] Boschi A., Uccelli L., Marvelli L., Cittanti C., Giganti M., Martini P. (2022). Technetium-99m Radiopharmaceuticals for Ideal Myocardial Perfusion Imaging: Lost and Found Opportunities. Molecules.

[84] Gould K.L., Taegtmeyer H. (2004). Myocardial ischemia, fluorodeoxyglucose, and severity of coronary artery stenosis: The complexities of Curr Cardiol Rep (2010) 12:170–178 177 metabolic remodeling in hibernating myocardium. Circulation.

[85] Jain D., Ghanbarinia A., He Z.X. (2009). Developing a new PET myocardial perfusion tracer. J. Nucl. Cardiol..

[86] Curley D., Lavin Plaza B., Shah A.M., Botnar R.M. (2018). Molecular imaging of cardiac remodelling after myocardial infarction. Basic Res. Cardiol..

[87] Maron D.J., Hochman J.S., Reynolds H.R., Bangalore S., O’Brien S.M., Boden W.E., Chaitman B.R., Senior R., López-Sendón J., Alexander K.P. (2020). Initial Invasive or Conservative Strategy for Stable Coronary Disease. N. Engl. J. Med..

